# The Use of Cognitive Tests in the Assessment of Dyslexia

**DOI:** 10.3390/jintelligence11050079

**Published:** 2023-04-26

**Authors:** Nancy Mather, Deborah Schneider

**Affiliations:** 1 Department of Disability and Psychoeducational Studies, University of Arizona, Tucson, AZ 85701, USA; 2 Hoeft BrainLENS Laboratory, University of Connecticut, Storrs, CT 06268, USA

**Keywords:** dyslexia, reading disorder, cognitive assessment, intelligence tests, phonological awareness, linguistic risk factors

## Abstract

In this literature review, we address the use of cognitive tests, including intelligence tests, in the assessment and diagnosis of dyslexia, from both historic and present-day perspectives. We discuss the role of cognitive tests in the operationalization of the concepts of specificity and unexpectedness, two constructs considered essential to the characterization of dyslexia since the publication of early case reports in the late nineteenth century. We review the advantages and disadvantages of several approaches to specific learning disabilities’ identification that are used in schools. We also discuss contemporary debates around the use of standardized cognitive testing in dyslexia evaluations, in particular, the arguments of those who favor an approach to diagnosis based on prior history and the results of a comprehensive evaluation and those who favor an approach based on an individual’s response to intervention. We attempt to explain both perspectives by examining clinical observations and research findings. We then provide an argument for how cognitive tests can contribute to an accurate and informed diagnosis of dyslexia.

## 1. Introduction 

Dyslexia, the most common specific learning disability, affects individuals’ abilities to read and spell and is influenced by multiple neurobiological, genetic, and environmental factors (Fletcher et al. 2019; Peterson and Pennington 2012, 2015). Prevalence estimates for dyslexia range from 7% of the global population (Yang et al. 2022) to 20% of the school-age population (Shaywitz et al. 2021). Despite the variation in estimates, dyslexia is a common and significant challenge for many individuals and their families. As such, the accurate diagnosis and effective treatment of dyslexia are of considerable importance to a large subsection of the population. 

Cognitive tests have played a central role in the assessment of dyslexia for nearly a century (Monroe 1932). Schneider and Kaufman (2017) explained that the term cognitive assessment refers not only to the use of intelligence (IQ) tests but also to any test that is designed to identify cognitive processing deficits that can influence the development of academic skills (p. 8). In this article, we address the use of cognitive tests, including intelligence tests, in the diagnosis of dyslexia, from both historic and present-day perspectives. We also discuss contemporary debates around the use of standardized cognitive testing in dyslexia, in particular, the arguments of those who favor an approach to diagnosis based on the assessment profile of an individual and those who favor an approach based on an individual’s response to intervention. We attempt to explain both perspectives by examining both clinical observations and research findings. We then provide an argument for the usefulness of cognitive tests in the diagnosis of dyslexia.

Two concepts, specificity and unexpectedness, have traditionally been operationalized through the use of both cognitive and achievement tests. They have long been considered to be fundamental to the diagnosis of dyslexia in particular and specific learning disabilities (SLD) in general (Grigorenko et al. 2020). In the case of dyslexia, specificity refers to the notion that the difficulties in reading and spelling are not accompanied by significant deficits in other academic areas (Peterson and Pennington 2015). Unexpectedness refers to the idea that individuals with dyslexia experience significant difficulties in reading that cannot be fully explained by their intelligence or environmental factors alone (Shaywitz and Shaywitz 2020). These two concepts have been associated with dyslexia since the late nineteenth century.

## 2. Historic Perspective 

The concepts of specificity and unexpectedness are not novel by any means. They are documented in case studies dating from the late nineteenth and early twentieth centuries. During this period, individuals who experienced significant difficulty in learning to read or lost the ability to read were characterized as suffering from word blindness. Notably, these individuals were often described as generally intelligent. As an example, Adolph Kussmaul, a German neurologist, documented the case of an adult patient with severe word blindness in 1877, noting that “a complete text blindness may exist although the power of sight, the intellect, and the powers of speech are intact” (Kussmaul 1877, p. 595). Similarly, in 1896, W. Pringle Morgan, a British eye surgeon, described a fourteen-year-old boy named Percy F., who struggled to learn to read but had no difficulty with arithmetic and was considered “…bright and of average intelligence in conversation.” Morgan noted that the schoolmaster believed the boy would be the smartest lad in the school if the instruction were entirely oral (Morgan 1896, p. 1378). Hinshelwood, a Scottish ophthalmic surgeon practicing in the early twentieth century, described children with *congenital word blindness* as having a good memory and average or above-average intelligence in other respects. In fact, he noted that many parents reported that their children with word blindness were, apart from their difficulty with reading, the most intelligent members of their families (Hinshelwood 1902, 1917). 

Travis (1935), likewise, described cases of special disability, in which subjects manifested a discrepancy between general intelligence and achievement in specific subjects. These children often exhibited discrepancies in closely correlated areas, such as reading and verbal intelligence. He noted that a child with intact cognitive abilities who cannot read but can comprehend text that is read aloud should be considered to have a *special disability*, clearly articulating the concepts of specificity and unexpectedness and their importance in identifying individuals with dyslexia. 

One of the first formal methods designed to capture the unexpectedness of specific reading difficulties was to compare intelligence test results with reading performance through the use of a discrepancy procedure. In her classic book *Children Who Cannot Read*, Monroe (1932) described the use of intelligence tests to identify children with specific reading disabilities. She observed that the reading defects “…may occur at any intellectual level from very superior to very inferior, as measured by intelligence tests” (p. 6). She proposed that intelligence tests be used as part of the diagnostic process to exclude difficulties caused by more global deficits and ensure the specificity of the disability. More precisely, she designed a *reading index*, a diagnostic procedure in which a child’s reading level was compared with their performance on tests of arithmetic and their mental age (obtained from the Stanford revision of the Binet–Simon tests (Terman et al. 1917)) and the mean level of reading achievement among age peers. In this way, it was possible to discriminate between children with dyslexia (i.e., those with a specific reading disability affecting a narrow range of skills) and those with more global difficulties in cognitive abilities, achievement, or both. Monroe’s reading index was likely the first documented use of an ability–achievement discrepancy procedure to identify a specific reading disability.

Despite its utility, problems with the use of a discrepancy procedure were noted early on. Monroe and Backus (1937) observed that children with generally intact cognitive abilities may demonstrate weaknesses in specific skills measured by intelligence tests, thereby depressing their overall scores and reducing the probability of detecting a discrepancy. Likewise, Orton (1925) remarked that intelligence tests may yield “an entirely erroneous and unfair estimate of the intellectual capacity of [word blind] children” (p. 582). These early observations are supported by more recent research that demonstrates how dyslexia has a clear and cumulative effect on intelligence test scores, as reading difficulties can impede vocabulary acquisition and the development of verbal ability, which are two important components of many intelligence tests (Ferrer et al. 2010; Meyer 2000).

## 3. Current Identification Guidelines in Diagnostic Manuals

In the ninety years since the publication of Monroe’s seminal work, the use of the ability–achievement discrepancy procedure has continued to be the subject of intense debate (Catts and Petscher 2021), and several alternative diagnostic methods have been developed. Nevertheless, the concepts of specificity and unexpectedness continue to form the core of the definition of dyslexia, and the basic principle of comparing reading achievement with performance in other areas remains central to diagnosis. Kavale and Forness (2000) explained that the word *specific* in the term specific learning disability was clearly intended to indicate that the affected individual had a limited number of underlying deficits. Moreover, the diagnosis of dyslexia continues to require the exclusion of other factors that might better explain the reading difficulties, such as global deficits, inadequate instruction, or insufficient mastery of the language of instruction. For example, individuals with low global intelligence or an intellectual disability may have reading difficulties, but their impairments are not considered unexpected given their overall cognitive abilities. Similarly, individuals with vision or hearing impairments may also experience difficulties with reading, but their impairments are not specific to reading and are instead related to their sensory disabilities. In these cases, the difficulties are expected, and interventions are selected that are appropriate to their etiology. With dyslexia, however, the reading difficulties are viewed as unexpected given the overall abilities and experiences of the individual (Ferrer et al. 2010; Shaywitz and Shaywitz 2020). An overview of contemporary approaches to the definition and diagnosis of dyslexia follows. 

Within clinical practice, the diagnostic criteria for SLD in reading are well defined. Most practitioners refer to the criteria outlined in major diagnostic manuals, such as the American Psychiatric Association’s (APA) *Diagnostic and Statistical Manual of Mental Disorders*, 5th Edition (DSM-5-TR; APA 2022), and the World Health Organization’s (WHO) *International Classification of Diseases,* 11th Revision (ICD-11; WHO 2022), when making diagnostic decisions. The criteria included in each of these manuals are remarkably similar, both providing guidelines for diagnosis that target the concepts of specificity and unexpectedness and each acknowledging the neurobiological basis of the disorder and the multifactorial contributors to its severity and presentation. 

### 3.1. DSM-5-TR

According to the DSM-5-TR, specific learning disorder (SLD) is characterized by difficulties learning and using academic skills (APA 2022). The diagnosis of SLD requires the presence of persistent difficulties in one or more areas of academic achievement that are not better explained by intellectual disability, sensory impairments, neurological or medical conditions, lack of opportunity to learn, or environmental factors. Per the DSM-5-TR, dyslexia falls under the category of *specific* (emphasis added) learning disorder with impairment in reading, which is characterized by difficulties with accurate or fluent word recognition, poor reading comprehension, and/or poor reading speed. The DSM-5-TR requires that the difficulties must have persisted for at least 6 months despite targeted intervention and support and must be diagnosed based on a comprehensive assessment that includes the use of standardized tests and clinical observations. Moreover, achievement in the area(s) of difficulty must be significantly below what is expected for the child’s age, overall intelligence, and educational level, satisfying the criterion of unexpectedness, and the child’s difficulties must be found to interfere with academic achievement and/or daily activities.

Consistent with statutory guidelines for the identification of dyslexia in the United States, the DSM-5-TR emphasizes the need for a multidisciplinary approach to the diagnosis of SLD, involving collaboration between educators, psychologists, and other professionals. Likewise, the DSM-5-TR requires the consideration of multiple sources of data, of which standardized testing is only one piece. Finally, while it does not require the use of an ability–achievement discrepancy procedure per se, it does clarify that a discrepancy between the child’s achievement and what would be expected given their age and intelligence is necessary for diagnosis and that a diagnosis of intellectual disability is generally inconsistent with a diagnosis of dyslexia. 

### 3.2. ICD-11

While the guidelines outlined in DSM-5-TR and the ICD-11 are remarkably similar, some differences exist between the two. Specifically, the WHO’s diagnostic criteria strongly recommend, but do not require, the use of standardized testing, acknowledging that such testing may not always be feasible (WHO 2022). Moreover, the ICD-11 does not use the term *specific learning disorders*, instead characterizing these learning difficulties as *developmental learning disorders* (DLDs), which comprise a group of neurodevelopmental disorders that affect a child’s ability to acquire and use skills related to reading, writing, mathematics, and/or related academic skills. 

The ICD-11 describes dyslexia as a developmental learning disorder with impairment in reading (DLD-R), which is characterized by difficulties in word recognition, decoding, reading accuracy, and/or fluency, satisfying the criterion of specificity of the disorder. As with the DSM-5-TR, the ICD-11 specifies that difficulties must have been present for at least 6 months despite appropriate instruction and that the difficulties must significantly interfere with academic achievement or activities of daily living. Similarly, it satisfies the criterion of unexpectedness, stating that difficulties must not be better explained by other factors, such as sensory impairment, disorder of intellectual development (intellectual disability), inadequate instruction, or social or cultural adversity. In addition to the above criteria, the ICD-11 also requires that the diagnosis should be made based on a comprehensive evaluation involving multiple sources of information, of which standardized testing should *ideally* be one component (WHO 2022).

## 4. Examples of Current Definitions

Despite the fact that nearly 150 years have elapsed since word blindness (dyslexia) was first described in the literature, strong consensus has yet to be achieved concerning a clear, useful definition of the disorder (Tønnessen 1997). Whereas numerous professional organizations throughout the world have developed definitions of dyslexia, no universally accepted definition exists. Nevertheless, the concepts of specificity and unexpectedness are common among most definitions. The most commonly used definition of dyslexia in the United States is that of the International Dyslexia Association (IDA).

### 4.1. International Dyslexia Association

In their definition, the IDA highlights both the specificity and unexpectedness of dyslexia, describing the disorder as “…a *specific* [emphasis added] learning disability that is neurobiological in origin. It is characterized by difficulties with accurate and/or fluent word recognition, poor spelling, and decoding abilities. These difficulties typically result from a deficit in the phonological component of language that is often *unexpected* [emphasis added] in relation to other cognitive abilities and the provision of effective classroom instruction. Secondary consequences may include problems in reading comprehension and reduced reading experience, which can impede the growth of vocabulary and background knowledge” (IDA 2002). 

While otherwise consistent with the conception of dyslexia provided in legislation and major diagnostic manuals, the IDA definition of dyslexia nevertheless has certain limitations, in that it focuses almost exclusively on the role of phonological skills in dyslexia and does not include other cognitive correlates of the disorder (nor does it address genetic (Parachchini et al. 2007), epigenetic (Theodoridou et al. 2021), or environmental contributions to the disorder). Ample research indicates that verbal abilities, phonological processing, rapid automatized naming, orthographic processing, working memory, and processing speed are all linguistic risk factors that are associated with dyslexia (Brady 2019; Fuchs et al. 2011; Georgiou et al. 2021; Mather and Jaffe 2021; Pennington et al. 2019; Schneider and Kaufman 2017; Warmington and Hulme 2012). 

### 4.2. British Dyslexia Association (BDA)

In contrast to the IDA definition, the definition adopted by the BDA clarifies that multiple cognitive risk factors are characteristic of dyslexia. Specifically, the BDA has adopted the Rose (2009) definition of dyslexia, stating, “Dyslexia is a learning difficulty that primarily affects the skills involved in accurate and fluent word reading and spelling. Characteristic features of dyslexia are difficulties in phonological awareness, verbal memory and verbal processing speed. Dyslexia occurs across the range of intellectual abilities. It is best thought of as a continuum, not a distinct category, and there are no clear cut-off points. Co-occurring difficulties may be seen in aspects of language, motor co-ordination, mental calculation, concentration and personal organisation, but these are not, by themselves, markers of dyslexia. A good indication of the severity and persistence of dyslexic difficulties can be gained by examining how the individual responds or has responded to well-founded intervention.” The BDA definition captures the specificity of the disorder in its description of the cognitive profile of individuals with dyslexia, a profile that is characterized by weaknesses in several language-related processes, as opposed to the IDA definition, which refers to phonological processes alone. It also makes reference to disorders most commonly comorbid with dyslexia, while clarifying that they are not in themselves markers of dyslexia. In contrast with the IDA definition, however, it de-emphasizes the concept of unexpectedness, specifying that dyslexia can occur across a range of intellectual abilities.

### 4.3. The First Step Act of 2019

A more recent definition of dyslexia was presented in the United States in the First Step Act of 2019. This definition explicitly refers to the concepts of specificity and unexpectedness. The First Step Act of 2019 defines dyslexia as “…an unexpected difficulty in reading for an individual who has the intelligence to be a much better reader, most commonly caused by a difficulty in phonological processing (the appreciation of the individual sounds of spoken language), which affects the ability of an individual to speak, read, and spell” (18 USC § 3635[1]). That said, the language of the act raises questions about how an evaluator must document the unexpectedness of the reading disability. In particular, the act appears to mandate the use of an intelligence test as part of the identification criteria. Although consensus exists that unexpected underachievement is the core of SLD, controversy exists regarding how to operationalize this unexpectedness (Grigorenko et al. 2020). Moreover, considerable disagreement exists within the field regarding the need for and utility of data from intelligence tests. 

## 5. Individuals with Disabilities Education Improvement Act of 2004 (IDEA 2004)

In the United States, the identification of dyslexia within schools is controlled by the regulations defined in the Individuals with Disabilities Education Improvement Act of 2004 (IDEA 2004). Under IDEA 2004, dyslexia falls within the category of specific learning disorder (SLD). The SLD category comprises eight areas of learning difficulty, for which affected students are entitled to service provision and an individualized education program: oral expression, listening comprehension, written expression, basic reading skills, reading fluency skills, reading comprehension, mathematics calculation, and mathematics problem solving. In the context of IDEA 2004, dyslexia is classified as an SLD in basic reading skills or reading fluency. 

In contrast to the First Step Act definition, IDEA 2004 states that “a local educational agency shall not be required to take into consideration whether a child has a *severe* [emphasis added] discrepancy between achievement and intellectual ability” when determining eligibility for services under IDEA 2004; moreover, 20% of states explicitly prohibited the use of an ability–achievement discrepancy procedure as of 2015 (Maki et al. 2015). IDEA 2004 further states that no single measure may be used as the sole criterion for determining eligibility for special education services. Instead, it emphasizes the importance of a comprehensive evaluation that includes multiple measures and data sources to determine whether a child has a learning disability. The evaluation should include data on cognitive, academic, and behavioral performance, as well as other relevant factors, such as medical and developmental histories (Hale and Fiorello 2004).

IDEA 2004 outlines the following three procedures that may be used in SLD identification: (1) the identification of a significant discrepancy between intellectual ability and achievement; (2) the use of other alternative research-based procedures, most often operationalized as a pattern of strengths and weaknesses (PSW) approach; and (3) a student’s response to evidence-based intervention, often referred to as response to intervention (RtI). Whereas the first two procedures typically include data from standardized tests, including tests of intelligence, the RtI process often does not. Instead, RtI adopts a definition of SLD that stands in contrast with those outlined in major diagnostic manuals. Likewise, the process for identification within the RtI framework does not necessarily require the use of standardized instruments, which have always been considered essential to a comprehensive dyslexia evaluation. The next section describes and examines these three procedures for use in dyslexia evaluations.

### 5.1. Ability–Achievement Discrepancy Procedure

The ability–achievement discrepancy procedure was designed to capture the concept of unexpectedness in the diagnosis of SLD, typically requiring the detection of a statistically significant discrepancy between a child’s reading achievement and their performance on a test of intelligence or cognitive abilities. To determine whether a significant discrepancy exists, several procedures can be used; however, the most common method is to calculate the difference between the child’s full-scale IQ score and their scores on a standardized test of academic achievement (e.g., reading, writing, or math). The size of the discrepancy required for diagnosis varies, but a discrepancy of one to two standard deviations (i.e., 15–30 points on the most commonly used standardized instruments; Meyer 2000) is typical.

The ability–achievement discrepancy procedure has certain strengths. In particular, it clearly addresses the diagnostic criterion of unexpectedness specified in widely used diagnostic manuals, and if used in conjunction with comprehensive achievement testing, it addresses the criterion of specificity as well. Moreover, it provides a standardized method for identifying learning disorders, and the data derived from cognitive testing can be used to exclude or diagnose other disorders as well. 

Despite its widespread use in the diagnosis of SLD (Benson et al. 2020), this approach also has several serious limitations. The use of this procedure has been described as a wait-to-fail approach (Meyer 2000), as students typically have to fall quite a bit behind their peers in order to exhibit a discrepancy large enough for diagnosis. In addition, arbitrary cut-offs for the magnitude of the discrepancy may serve to deny struggling readers access to services. Finally, the use of a discrepancy model for the early identification of dyslexia often results in what Ozernov-Palchik and Gaab (2016) have described as the *dyslexia paradox*: a child is unlikely to demonstrate a discrepancy of sufficient magnitude for diagnosis in the first two years of school when identification and intervention would be most beneficial. By using measures of phoneme awareness and letter knowledge coupled with information from family history, by contrast, it is possible to predict risk for dyslexia at the beginning of kindergarten (Pennington et al. 2019). Phonological awareness tests predict achievement, and that can help identify children who are at risk for a reading disability (Fletcher and Miciak 2017).

Another important limitation involves the effects of dyslexia on the measurement of intelligence. In typically developing readers, a strong positive relationship exists between intelligence test scores and reading ability; however, for readers with dyslexia, this relationship proves much weaker due to reduced exposure to reading material and consequent deficits in the acquisition of vocabulary and general knowledge (Ferrer et al. 2010; Meyer 2000). Moreover, group differences in both IQ and reading skills between typical readers and those with dyslexia tend to increase over time, with reading difficulties negatively affecting reading, language, and overall cognitive development. As such, the likelihood of detecting a significant discrepancy diminishes with time.

In a study conducted by Tanaka et al. (2011), 131 children with poor reading ability were examined, including those with high IQ scores (discrepant readers) and those with low IQ scores (nondiscrepant readers). Both discrepant and nondiscrepant poor readers exhibited similar patterns of reduced activation in the left parietotemporal and occipitotemporal regions, implying that poor readers, regardless of their IQ, experienced similar difficulties in relation to phonological processing. The results of this study provide further support for the view that the ability–achievement discrepancy model may not be the best method for identifying dyslexia and that alternative models of diagnosis, such as a PSW approach, may be more sensitive (Hale et al. 2010), whereas multitiered models of identification and service provision (i.e., RtI) may be more inclusive (Miciak and Fletcher 2020).

### 5.2. Alternative Research-Based Procedures (Pattern of Strengths and Weaknesses)

The PSW approach is the most commonly used alternative research-based procedure for the identification of dyslexia and SLD permitted under IDEA 2004. The PSW approach arose out of clinical observations concerning specificity and the unique cognitive profiles of children with SLD, in particular, dyslexia. As an example, nearly 60 years ago, Gallagher (1966) described children with *developmental imbalances*, characterized by a significant pattern of strengths and weaknesses among their cognitive abilities. These children had average or above-average functioning in some domains and well below-average functioning in others. In psychological reports describing children with dyslexia, it is fairly common to see statements explaining that a full-scale IQ score could not be validly derived, as the child demonstrated an unusual level of variability in performance from index to index. It is now well established that such *imbalanced* cognitive profiles are characteristic of children with dyslexia, as well as other specific learning disabilities (Hale et al. 2010). 

The PSW method is an approach to diagnosing SLD that involves a comprehensive evaluation of skills across various domains, including cognitive abilities, academic achievement, attention, and/or language, to identify patterns of strengths and weaknesses that are characteristic of SLD (Schultz et al. 2012). The PSW approach was designed to establish both logical and empirical links between a child’s cognitive profile and academic achievement, particularly in areas of concern (Fiorello and Primerano 2005; Flanagan et al. 2006, 2013). It is premised on the empirically supported idea that individuals with SLD often have a unique pattern of cognitive strengths and weaknesses that differs from individuals without learning disabilities (Kavale and Forness 2000). Like the ability–achievement discrepancy procedure, the PSW approach captures the concept of unexpectedness; however, it does so by comparing intact cognitive abilities with those affected by the SLD as opposed to comparing overall intelligence with achievement, thus avoiding one of the principal weaknesses of the ability–achievement discrepancy procedure as previously discussed. Moreover, it captures the concept of specificity, providing a comprehensive portrait of an individual’s strengths and weaknesses across numerous areas of cognitive and academic functioning and identifying patterns of performance typical of SLD.

The PSW assessment process includes a comprehensive evaluation of cognitive abilities, academic skills, and other factors that may affect learning, such as attention and motivation. The evaluation may include tests of intelligence, academic achievement, attention, memory, language, and other cognitive abilities (Hale and Fiorello 2004). Following an assessment, an evaluator must determine whether the child exhibits a pattern of strengths and weaknesses that is consistent with a diagnosis of SLD. 

Some practitioners use a diagnostic algorithm, such as the cross-battery approach (XBA; Flanagan et al. 2013), the concordance–discordance model (CDM; Hale and Fiorello 2004), the discrepancy–consistency model (DCM; Naglieri and Feifer 2020), the core-selective evaluation process (C-SEP; Schultz and Stephens 2015), or the dual-discrepancy/consistency model (DD/C; Flanagan et al. 2018). It is important to emphasize, however, that these algorithms are designed to facilitate the PSW process; they are not PSW approaches in themselves, and they should not be conflated with the basic concept of a PSW approach. Others apply a PSW approach by looking at specific theoretical models of intelligence and how they relate to school achievement. For example, using modern network intelligence theories to explore cognitive abilities, McGrew et al. (2023) described the relevance of Cattell–Horn–Carroll (CHC) broad ability scores to understanding school achievement. Still other practitioners do not employ such methods; they select specific instruments based on the referral question and use their knowledge of testing instruments and clinical judgment to plan an evaluation, consider a diagnosis, and derive treatment implications. In every case, however, the PSW approach involves the analysis and synthesis of multiple sources of information, as well as the application of clinical judgment and discretion.

The PSW approach has several advantages over traditional discrepancy-based models for diagnosis. Unlike discrepancy-based models, which rely on a simple comparison between IQ and achievement to identify SLD, the PSW approach involves the analysis of the unique pattern of cognitive strengths and weaknesses specific to the individual (Kavale and Forness 2000). By identifying these patterns, the PSW approach can improve the specificity of diagnosis and help identify the specific areas in which an individual may need support (Hale et al. 2010). The PSW approach can also provide valuable information for treatment planning, as interventions can be tailored to an individual’s specific areas of cognitive strengths and weaknesses (McCloskey et al. 2012). The educational plan may include academic accommodations and interventions designed to support the individual’s specific learning needs. 

A potential weakness of the PSW approach is that it may be more difficult to implement in some settings, such as schools with limited resources or lack of personnel with expertise in cognitive assessment. Likewise, a PSW evaluation can be time consuming and resource intensive (Miciak et al. 2018). The comprehensive assessment and analysis required to identify patterns of strengths and weaknesses may take several hours, and the development of an individualized intervention plan may require ongoing support and resources. A recent analysis, however, has challenged the view that PSW evaluations are overly burdensome, concluding that high-quality, individualized assessment is not nearly as resource intensive as has been asserted by critics (Shanock et al. 2022).

Finally, little evidence supports the conclusion that the data generated from PSW evaluations are routinely used to tailor instruction provided to students with SLD (Elliott and Resing 2015), so this particular advantage of the PSW model may be stronger in theory than in practice. Likewise, while the PSW approach makes intuitive sense, the research base on the diagnostic sensitivity remains thin, at least with respect to research in which the model has been used in a manner consistent with the guidelines provided by the authors of various PSW algorithms. Moreover, the use of PSW methods for SLD identification in the United States is increasing (Benson et al. 2020) despite concerns regarding the psychometric properties of instruments routinely used in PSW procedures, as well sensitivity and specificity of common PSW algorithms (Maki et al. 2022). For example, Beaujean et al. (2018) raised the following three concerns about the DD/C model: (a) The dual discrepancy/consistency model requires test scores to have “properties that they fundamentally lack.” Specifically, the model assumes that test scores are normally distributed and free of measurement error, but in reality, test scores often have skewed distributions and are subject to measurement error. This increases the probability of misidentification. (b) There are insufficient experimental data supporting the use of the dual discrepancy/consistency model. This means that there is little evidence to suggest that the model actually improves the identification of SLD compared with other methods. (c) There is evidence supporting flaws in the dual discrepancy/consistency model of SLD identification. Specifically, the model relies too heavily on cognitive test scores that may not be reliable indicators of SLD in reading. Concerns have also been raised regarding the accuracy of the factor structures described within tests and the influence of general intelligence across the tests within a battery, complicating clinical interpretation (McGill et al. 2018). Given these concerns, continued research in this area is needed.

### 5.3. Response to Intervention

Response to intervention (RtI) is a three-tiered system of interventions that can be used to identify children who may have specific learning disabilities, such as dyslexia (Kavale and Forness 2000). Within the RtI framework, the concept of unexpectedness is operationalized as the persistence of difficulty in learning to read despite the provision of evidence-based reading instruction (Miciak and Fletcher 2020). Some proponents of the model assert that within the RtI framework, cognitive testing is unnecessary for the diagnosis of reading-related learning disabilities, arguing that dyslexia should be characterized by persistent difficulty in learning to read rather than an underlying cognitive condition of neurobiological origin (Catts and Petscher 2021).

The RtI model involves three tiers of intervention, each of which provides increasingly intensive support to students who continue to struggle academically. In Tier 1, all students receive high-quality, evidence-based instruction in general education settings. In Tier 2, targeted, research-supported interventions are provided to students who are at risk for reading difficulties. If students still do not make adequate progress, they move to Tier 3, where they receive more intensive and individualized interventions and may be referred for a special education evaluation (Fuchs and Fuchs 2006).

The RtI approach uses frequent progress monitoring to track a student’s response to intervention. If a student does not respond to high-quality, evidence-based intervention, then a multidisciplinary team may refer the student for an individual evaluation to determine eligibility for special education services under the SLD category (Fletcher and Vaughn 2009). During an individual evaluation for SLD within the RtI framework, RtI data are considered alongside other sources of information, including academic, behavioral, and/or cognitive assessments. This process incorporates the collection of data across multiple domains, such as reading, writing, and mathematics, to determine the extent of the student’s learning difficulties and exclude other potential causes of the student’s difficulties (Fletcher and Miciak 2017).

One of the main strengths of the RtI framework is its emphasis on providing early and targeted interventions for all struggling readers, which can prevent the emergence of more significant reading difficulties in the future (Miciak and Fletcher 2020). Furthermore, the RtI framework is more inclusive, as it does not require an IQ–achievement discrepancy to identify a child who may have dyslexia. Moreover, the cognitive profiles of children who fail to respond to reading intervention within an RtI framework have been found to mirror closely those of those with reading-related learning disorders (Peterson et al. 2021), leading advocates of RtI to argue that testing for cognitive abilities is unnecessary in most cases. Likewise, the reading-related prognosis of nonresponsive poor readers are often comparable whether or not they have an IQ–achievement discrepancy (Wagner 2008). There is also a strong equity-based argument to be made that all poor readers can benefit from the intensive, individualized instruction provided to students with dyslexia, as reading is a skill essential to informed participation in broader society. Thus, RtI is useful for reducing the impact of a disability, as well as early intervention for children at risk for reading failure (Decker et al. 2013). 

An important limitation of the RtI framework, however, is the lack of consensus on procedures for the identification of students who are not responding to intervention, leading to potential delays in the diagnosis of SLD (Reynolds and Shaywitz 2009). Likewise, there is a lack of standardization in the RtI assessment process (McKenzie 2009), meaning that different schools and education authorities may have broadly varying approaches to identification and qualification under the SLD category (Hudson and McKenzie 2016), and as with the ability–achievement discrepancy procedure, many struggling students fail to meet cut points for service provision (Balu et al. 2015). Others have criticized the RtI approach for undermining the integration of students with learning disorders into the general education classroom, in that it leans heavily on potentially noninclusive models of instruction for nonresponsive learners (Ferri 2012).

Finally, research in real-world settings has revealed serious limitations in the efficacy of RtI programs, finding insignificant or even negative effects on achievement, especially in the early grades (Balu et al. 2015). In fact, a large federal analysis of the use of RtI in American public schools found that RtI, as currently implemented, may actually harm many of the students it is intended to help: “To summarize, results [...] show that early-grade elementary students *at the margin* of being considered at risk by current screening measures failed to benefit from Tier 2 or Tier 3 intervention services provided to them. In first grade, these students actually fell further behind their counterparts who, because they scored just above the cut point on the screening variable for intervention, were placed to receive only Tier 1 services” (Balu et al. 2015, p. 93) and that “on average, the estimated impact [of the use of RtI programs in the schools studied] is negative or not statistically significant” (Balu et al. 2015 p. 100). 

The RtI approach is not a diagnostic procedure in itself but a framework for identifying and providing support to struggling learners. According to the Office of Special Education Programs (OSEP 2011), RtI cannot be used to deny an individual evaluation to a child who is suspected of having a learning disorder, nor can an evaluation be delayed on the basis that a child has not participated in an RtI program. Additionally, the United States Department of Education (DOE 2006, p. 46648) has clarified that an “RTI process does not replace the need for a comprehensive evaluation. A public agency must use a variety of data gathering tools and strategies even if an RTI process is used. The results of an RTI process may be one component of the information reviewed as part of the evaluation procedures … and an evaluation must include a variety of assessment tools and strategies and cannot rely on any single procedure as the sole criterion for determining eligibility for special education and related services.” Likewise, data gathered from RtI alone are likely insufficient for diagnosis consistent with common diagnostic criteria, such as those outlined in the DSM-5-TR or ICD-11. Nevertheless, the data generated by the RtI process can inform decision making about whether further evaluation is necessary and be used in conjunction with other data and assessments to determine eligibility for special education services (Feifer 2008). 

## 6. Arguments against the Use of Cognitive Tests in the Assessment of Dyslexia

Historically, intelligence testing has been an essential component in the assessment and identification of SLD, but a growing chorus of experts have asserted that cognitive tests may not be the best tool for this purpose (Miciak et al. 2014; Miciak et al. 2016; Miciak and Fletcher 2020; Taylor et al. 2017). Ultimately, their arguments are premised on a rejection of the conception of specific learning disorders as defined in major diagnostic manuals, such as the DSM-5-TR and ICD-11(i.e., definitions requiring the demonstration of specificity and unexpectedness with a high degree of certainty). Instead, they advocate for a more expansive model of learning disorder, implying that any persistent failure to respond to evidence-based reading instruction should be characterized as an SLD, provided that no exclusionary factors are identified (Miciak and Fletcher 2020). 

The appeal of this model is easy to understand, as it extends the provision of services under the SLD category even to those students whose profiles would not satisfy diagnostic criteria for specific learning disorders using common algorithms (e.g., ability–achievement discrepancy or the PSW approach) or diagnostic criteria (e.g., DSM-5-TR or ICD-11; APA 2022; WHO 2022). Under federal law in the United States, the provision of services to children with specific learning disabilities is premised on the identification of a qualifying disability (IDEA 2004), so a more inclusive construction of disability results in a more inclusive model of service provision, thus ensuring that more children with persistent reading difficulties receive interventions and support consistent with an individualized educational program. 

That said, some advocates for the RtI approach argue for a conception of SLD that de-emphasizes the criteria of specificity and unexpectedness (insofar as these cannot be established with clinical precision absent the use of standardized measures), which have always been considered hallmarks of SLD, rather than explicitly calling for a model of service provision that provides special education to all children with persistent reading difficulties regardless of etiology. Some of the critiques of the use of standardized instruments in SLD diagnosis do not, however, appear to adequately represent how the PSW approach is commonly used in practice. As an example, some researchers have criticized the weak diagnostic specificity of a common PSW algorithm when used, absent clinical discretion, to “diagnose” specific learning disorders based on computer-generated test scores (Miciak et al. 2018); however, the algorithm in question relies upon evaluator discretion to establish the specificity of the disorder, using tests of achievement and other sources of data, as the algorithm does not include a criterion for a significant pattern of strengths and weaknesses among achievement areas. 

Similarly, researchers (Miciak et al. 2016) have criticized the predictive validity of certain algorithms, when applied to an assortment of test data purported to operationalize various Cattell–Horn–Carroll (CHC) theoretical categories; however, the data in question do not include complete core cognitive battery, nor any complete cluster or index within a cognitive battery. Although one measure of working memory^1^ is included, it is a subtest described by its coauthor as an evaluation of the central executive (i.e., a task of auditory attentional control, not primarily a task of the phonological loop) that “[c]hildren with dyslexia [seem to] score particularly well on” (Pickering 2006, p. 258)^2^. Still, others cite weaknesses in the diagnostic specificity of certain algorithms when applied—without clinical interpretation, discretion, or additional sources of information—to data obtained from an assortment of subtests^3^ derived from numerous instruments for which the algorithms were not optimized (Miciak et al. 2014). 

In routine practice, these algorithms would never be used in this manner and would be applied as part of a comprehensive evaluation, including, at a minimum, relevant core subtests from high-quality batteries of cognitive abilities and achievement as well as various other sources of data (e.g., data from standardized tests of reading-related cognitive abilities and reading abilities, school records, and individual history; Fiorello et al. 2014). Moreover, such head-to-head comparisons make little sense, even when performed using multiple sources of data derived from battery testing using gold standard instruments, as various PSW models, while conceptually similar, do not purport to employ equivalent procedures. For example, the DCM model requires a normative weakness in achievement, while the CDM does not; likewise, the DCM model requires a significant pattern of strengths and weaknesses among achievement areas, establishing specificity, while the CDM does not. Instead, the CDM model requires the clinician to establish specificity using other means. Finally, each of these models relies on the application of clinical interpretation and discretion to determine whether diagnostic criteria have been met. As noted by Miciak et al., this type of data simulation does not account for the role of clinical judgment. It stands to reason, therefore, that absent clinical interpretation and the consideration of various other sources of information, the diagnostic conclusions attributable to these models would differ, rendering such an evaluation of the validity of these algorithms essentially meaningless. Furthermore, these models offer uniform means by which to operationalize a PSW approach and evaluate data, but they are neither necessary nor sufficient in themselves to a PSW evaluation. 

Another criticism of the PSW approach relies on a conception of cognitive abilities as the product of general intelligence, represented by the psychometric *g* factor (Canivez et al. 2018), and/or relatively few underlying broad cognitive factors (Dombrowski et al. 2018). This conception, born of the application of bifactor statistical factor reduction techniques to cognitive testing data, is at variance with the eight-factor structure of the Cattell–Horn–Carroll (CHC)-based model of intelligence that informs many PSW approaches. Explicit in this criticism is the concern that the purported overfactorization of CHC-model-aligned instruments results in repeated measurement of the same underlying factors and consequently increases in the probability of a type I error (McGill et al. 2018). Whereas these arguments are appealing in that they provide a straightforward explanation for the large proportion of shared variance in measures of various cognitive abilities, they stand in contrast with hierarchical *g* (Carroll) or non-*g* (Horn) CHC-informed models of cognitive abilities (Keith and Reynolds 2010; McGrew et al. 2023; Schneider and McGrew 2018) and are at variance with the experience of those who employ those models in day-to-day-practice. To conflate factors as diverse as short-term memory, processing speed, fluid reasoning, and comprehension knowledge into a single construct would be anathema to most professionals working in the field, who use these factors routinely to specify the nature of students’ difficulties and to exclude more global deficits. Moreover, certain component factor analyses of the factor structure of the cognitive abilities measured by CHC-aligned instruments have supported both the broad abilities defined in the model, suggesting that CHC theory is a robust model for the measurement and description of cognitive abilities (Keith and Reynolds 2010; Schneider and McGrew 2018). Likewise, researchers performing psychometric network analyses of cognitive testing data have arrived at similar conclusions (McGrew et al. 2023) and have argued that the covariance among cognitive abilities should not be seen as an indication that all cognitive capacities are essentially due to a common cause (psychometric *g*) but that they all emerge as the products of a complex system of nonlinear dynamic interactions between biological and cognitive elements. Ultimately, these criticisms and their counterarguments are unlikely to be resolved anytime soon and fall beyond the scope of this article.

Other arguments of the critics of tests of cognitive abilities merit further examination as well. It is well established that individuals with reading-related learning disabilities have cognitive strengths and weaknesses that differ from those without disabilities (Thambirajah 2010); however, learning disabilities are not synonymous with general intellectual deficits (Kavale and Forness 2000), and IQ tests alone lack the sensitivity required for the accurate diagnosis of dyslexia. Many intelligence tests assess a broad range of cognitive abilities, including vocabulary, verbal and nonverbal reasoning, visuospatial skills, processing speed, and working memory; however, the reading difficulties experienced by students with dyslexia are generally related to specific weaknesses in phonological processing, rapid automatized naming, orthographic processing, processing speed, and verbal working memory (Cunningham et al. 2021; Georgiou et al. 2021; Moll et al. 2016; Vellutino et al. 2004). 

The most relevant of these abilities to dyslexia identification is phonological processing, which involves the ability to perceive and manipulate the sounds of language. Phonological processing deficits are a consistent hallmark of dyslexia (Pennington et al. 2019; Shaywitz and Shaywitz 2020). After implementing an intervention program with first graders, for example, Vellutino et al. (1996) concluded that many of the skills and abilities evaluated by intelligence tests are not as important for success in beginning reading as are phonological skills (p. 632). Phonological processing, however, is not measured on many commonly used intelligence tests; intelligence tests are designed to provide a valid and reliable picture of cognitive functioning across broad domains rather than a nuanced portrait of specific reading-related cognitive processes.

## 7. Arguments for the Use of Cognitive Tests in the Assessment of Dyslexia

The use of tests of cognitive abilities, including intelligence tests, can aid in the diagnosis and treatment of dyslexia by providing valuable information concerning a child’s overall cognitive profile, including both strengths and weaknesses (Hale et al. 2010). An analysis of this pattern can inform the diagnostic process and assist in the development of tailored supports, promoting improved response to intervention (Mascolo et al. 2014). Further, these tests can help rule in or rule out the existence of comorbid disorders and help identify twice-exceptional students with dyslexia. 

### 7.1. Identification of Comorbidity

In addition to aiding in the diagnosis of dyslexia, tests of cognitive abilities can be used to help identify comorbid conditions. High levels of comorbidity exist between dyslexia and other learning disorders. Dyslexia is commonly comorbid with disorders of attention, mathematics, and oral language (Snowling et al. 2020). In fact, approximately 50% of children with dyslexia will have an additional learning disorder (Moll 2022). 

For example, attention-deficit hyperactivity disorder (ADHD) is often comorbid with dyslexia (Mayes and Calhoun 2007), and the symptoms of ADHD can often mask and/or exacerbate the symptoms of dyslexia (Willcutt et al. 2007). Through the use of cognitive assessments, clinicians can often differentiate between the two disorders and suggest interventions to help address the symptoms of each, thus improving the overall efficacy of the treatment. Likewise, information from tests of cognitive ability can be used to distinguish dyslexia from other disorders that may impair reading achievement, such as a global developmental delay or intellectual disability. This is an important distinction, as the supports required by children with more global deficits tend to be far more comprehensive in nature and often include assistance with life skills development and adaptive behavior in addition to assistance with the acquisition of academic skills.

Whereas the concepts of specificity and unexpectedness have been under attack in recent years (e.g., Miciak et al. 2014, 2016; Taylor et al. 2017), they remain central to evaluation in conformity with the recommendations of major diagnostic manuals. Moreover, they remain useful insofar as they allow evaluators to distinguish among children who have deficits in a narrow range of skills and abilities but are otherwise typically developing from those who have global disabilities, children who have other disorders or comorbidities that can contribute to poor reading achievement (e.g., attention-deficit hyperactivity disorder (ADHD) or developmental language disorder (DLD)), and children who occupy the lower quartile of the distribution but do not meet the criteria for the diagnosis of a specific or global neurodevelopmental or language disorder. Appropriate treatment approaches, as well as the overall prognosis for these children, differ. 

The statutory definition of SLD in the United States requires that intellectual disability be excluded prior to diagnosis (Wodrich et al. 2006). As such, diagnostic methods must continue to include procedures and instruments designed to differentiate among different types of reading difficulty; the etiology of reading difficulty has obvious implications for both short-term intervention and long-term treatment planning. Moreover, a strong argument can be made that educators, families, and students have a right to know and understand the nature of developmental difficulties that are affecting a student’s reading development and progress (Shanock et al. 2022; Wodrich et al. 2006). The concepts of specificity and unexpectedness highlight the need for specialized assessments that help discriminate between dyslexia and other disorders. Ideally, these assessments would include measures designed to identify the weaknesses in phonological and linguistic processes involved in reading that characterize dyslexia (Peterson and Pennington 2012).

### 7.2. Identification of Twice-Exceptional Students

Intelligence test results can be useful for and are sometimes essential to the diagnosis of dyslexia. This is particularly the case in the identification of twice-exceptional students. The DSM-5-TR specifies that to be identified with a learning disorder, reading must be substantially below the mean for the individual’s chronological age in every case (Peterson and Pennington 2015). Thus, a twice-exceptional student might fail to meet diagnostic criteria; their reading scores may even fall within the average range when compared with peers despite being far below expectations given their overall intelligence. Shaywitz and Shaywitz (2020) stated, “There is no one single test score that ensures a diagnosis of dyslexia. It is the overall picture that matters. An extremely bright child who has a reading score in the average range but who struggles and cannot learn to read fluently… has dyslexia” (p. 166). 

In the case of twice-exceptional students, results from an IQ–reading discrepancy would be more appropriate than the DSM-5-TR criteria for identifying dyslexia (Pennington et al. 2019). In the United States, these children may receive a diagnosis of dyslexia, per the statutory definition of the disorder, even if a normative weakness is not present. Specifically, a United States statute defines dyslexia as “…an unexpected difficulty in reading for an individual who has the intelligence to be a much better reader …” (18 USC § 3635[1]) (retrieved from https://www.law.cornell.edu/uscode/text/18/3635, accessed on 15 February 2023). As such, twice-exceptional individuals with clinically significant reading problems, but who fail to meet the criterion of normative weakness, may be diagnosed using an IQ–achievement discrepancy procedure (Peterson and Pennington 2012, 2015) consistent with the guidelines outlined in IDEA (2004). The diagnosis of dyslexia in gifted students can be complicated, in that that their giftedness can mask their disability, and their disability can mask their giftedness (Baum and Owen 2004). Thus, for twice-exceptional students, cognitive testing is integral to both the definition and the diagnosis of dyslexia. Reading tests alone are insufficient because they fail to capture the discrepancy between the child’s potential and their actual achievement, thus denying them the intervention and support that could allow them to improve their reading and spelling and be far more successful in both school and work environments.

The patterns of strengths and weaknesses in the cognitive profiles of twice-exceptional students often resemble those of average-IQ students with dyslexia. In fact, phonological processing abilities can be quite independent of the level of intelligence (Shaywitz and Shaywitz 2020). In addition, the neurofunctional profiles of high- and average-IQ children with discrepancies between their IQ and reading achievement are often similar. A neuroimaging study demonstrated that the brains of high-IQ children with average single-word reading ability showed reduced activity in the exact same regions as average-IQ readers with dyslexia (Hancock et al. 2016). That is to say that despite average reading achievement, the brains of these children showed the same characteristics (left temporoparietal dysfunction believed to be associated with phonological processing) as those of poor readers. As such, there is a strong argument to be made that twice-exceptional children should be identified as having dyslexia and provided with remediation and support; the focus should be on the individual needs of the learner (Bell and Philippakos 2022).

## 8. Cognitive Correlates of Dyslexia: Considerations for Evaluation

A large body of research has documented the relationships between specific cognitive abilities and dyslexia (Shaywitz and Shaywitz 2005; Vellutino and Fletcher 2005; Vellutino et al. 2004). Identification of the specific cognitive and linguistic risk factors (e.g., poor phonological processing, slow rapid automatized naming, weak verbal working memory) can help explain the weaknesses in reading and spelling development. In general, students who have multiple cognitive deficits are at a much higher risk for dyslexia (Pennington et al. 2019). Therefore, comprehensive evaluation of these factors can help identify students at greatest risk and select and provide the interventions and accommodations most appropriate to their unique cognitive profiles (Decker 2008). 

Although two children may both have dyslexia, a reader who has weaknesses in phonological awareness and phonics requires a different type of intervention than a reader who has slow naming speed and reading rate. In the case of the former, a structured and intensive intervention would target phonological awareness and phonics skills; in the case of the latter, intervention would target word identification and fluency. Likewise, a student with dyslexia who has a weakness in working memory and struggles with both reading and math requires an intervention that addresses development of the skills and memory-related processes underlying both reading and math. In the subsequent section, we briefly discuss the evaluation of the cognitive correlates of dyslexia, including oral language abilities, phonological awareness, rapid automatized naming, and working memory. 

### 8.1. Oral Language

Studies have demonstrated that performance on measures of verbal ability, including vocabulary, listening comprehension, and verbal reasoning, is highly predictive of reading achievement (Mayes and Calhoun 2007). Measuring a child’s verbal ability using intelligence tests, therefore, can help identify or exclude weaknesses in verbal abilities common among children with reading-related learning difficulties. In addition, standardized tests of oral language provide clinicians with valuable information about a child’s language skills and can help differentiate among dyslexia, developmental language disorders, and other conditions. 

Several studies have supported the importance of oral language skills in dyslexia identification and intervention. In their review of the literature on cognitive resilience in dyslexia, Haft et al. (2016) found that strong oral language skills were associated with better reading outcomes, whereas lower oral language skills predicted poorer reading achievement. Similarly, in a study conducted by Nation and Snowling (2004), oral language skills were found to be strongly correlated with reading and writing abilities. The authors also reported that difficulties in oral language were a significant predictor of reading difficulties, suggesting the importance of addressing oral language in evaluations and interventions for dyslexia. In addition, verbal comprehension explains the strong relationship between reading and mathematics (Peterson et al. 2017).

Standardized tests of oral language can also provide valuable information about a child’s oral language comprehension, vocabulary, syntax, semantics, and pragmatics. These language components are essential for reading comprehension and written expression (Nation and Snowling 2004), and children with reading-related learning difficulties often have significant weaknesses in these areas (Adlof 2020; Catts et al. 2006; Snowling and Hulme 2021). Moreover, standardized tests of oral language can help clinicians identify comorbid conditions that can complicate the diagnosis and treatment of dyslexia; as noted, developmental language disorders are often comorbid with dyslexia (Catts et al. 2006; Moll 2022). They can also help identify strengths. Whereas phonological awareness is often impaired in individuals with dyslexia, the higher-level components of oral language are often intact (Shaywitz and Shaywitz 2020).

### 8.2. Phonological Awareness

Studies have consistently shown that children with dyslexia have difficulty with phonological awareness, which is the ability to recognize and manipulate the sounds of spoken language (Vellutino et al. 2004). Standardized tests that include phonological processing can provide clinicians with a reliable and valid measure of a child’s phonological processing skills. By assessing a child’s ability to identify and manipulate sounds and syllables, clinicians can determine whether weaknesses in phonological processing that are characteristic of dyslexia have contributed to the child’s difficulty in reading, increasing the specificity of the diagnosis and aiding in the selection of appropriate interventions. 

### 8.3. Rapid Automatized Naming

In addition to phonological awareness, RAN has been identified as another core deficit associated with dyslexia (Wolf and Bowers 1999, 2000). Phonological awareness and RAN are two salient but separable causes of reading impairment (Ozernov-Palchik et al. 2022). On RAN measures, children are presented with a card of familiar objects, letters, numbers, or colors, and asked to name them as quickly as they can. RAN is an important predictor of automatic word recognition and reading fluency (Araújo et al. 2015; Georgiou et al. 2016; Nelson 2015). In kindergarten and first grade, early naming speed deficits are good predictors of which children will struggle with reading fluency later in school (Wolf 2007). In addition, because RAN tasks do not require any reading, they may be administered to young children, making these tasks an easy way to help identify young children who are at risk for reading failure. These naming speed deficits persist into adolescence and adulthood (Denckla and Rudel 1976). RAN is an important linguistic risk factor for dyslexia in all languages and writing systems; thus, the inclusion of RAN tasks in both neuropsychological and educational assessments for dyslexia is strongly encouraged (Araújo and Faísca 2019). 

### 8.4. Working Memory

Working memory, the ability to hold and manipulate information in the mind in the service of learning and solving problems, is among the cognitive abilities most frequently impaired in children with dyslexia (Mayes and Calhoun 2007). Studies have shown that children with dyslexia often have weaker working memories compared with typically developing children (Gathercole and Alloway 2008) and that verbal working memory is a unique predictor of reading achievement (Dehn 2011; Stevenson et al. 2014). 

Likewise, intact working memory can mitigate deficits in achievement attributable to dyslexia. For example, van Viersen et al. (2016) found that gifted children with dyslexia but strong short-term memory, working memory, and oral language performed between students with dyslexia and typically developing children on reading tasks. An analysis of their cognitive profiles revealed typical weaknesses in phonological awareness and rapid naming. The authors concluded that although gifted children with dyslexia have linguistic risk factors common to many children with dyslexia, their intact working memory and strong oral language abilities help them compensate and may even mask their dyslexia. While debate still exists concerning the efficacy of working memory interventions, particularly with respect to the generalizability of skills acquired during such training (Melby-Lervåg and Hulme 2013), knowing whether an individual has strengths or weaknesses in oral language and working memory can influence the selection of accommodations and intervention planning. 

## 9. Conclusions

The main purposes of a dyslexia evaluation are to (a) determine if the individual has dyslexia, (b) specify the nature and degree of the underlying difficulties, (c) determine strengths, and (d) select appropriate accommodations and interventions. The reality is that the approaches for SLD identification that are outlined in major diagnostic manuals and IDEA 2004 are not ideal for the assessment of dyslexia. Although a PSW approach is perhaps most consistent with the concepts of specificity and unexpectedness, all of these approaches have weaknesses that, when used without clinical judgment, might potentially lead to the misidentification of individuals with and without dyslexia.

Research continues to enhance our understanding of the cognitive and linguistic risk factors that are implicated in dyslexia. Cognitive testing can provide valuable information about an individual’s verbal abilities and may assist in identifying specific strengths and weaknesses in language-related processes specifically implicated in dyslexia. Information obtained from traditional intelligence measures can also prove useful in establishing possible areas of strength (e.g., vocabulary, reasoning) that can help in the development of compensatory strategies. It is unnecessary to administer tests to measure all CHC factors; instead, evaluators should focus on those areas that are most relevant to the referral question and the age of the examinee, including the cognitive and linguistic risk factors most correlated with reading development and reading failure.

The assessment of dyslexia also requires evaluators who have the skill, insight, and discretion necessary to interpret the results. As Kilpatrick (2018) noted, “The practitioner’s greatest assessment tool is a strong knowledge base regarding the nature of typical word-reading development and the sources of reading difficulties” (p. 967). Whereas cognitive assessments can help evaluators address the what, the how well, and the why of dyslexia (Mather and Kaufman 2006), they cannot be used as the sole basis for a diagnosis. As with any other diagnosis, evaluators should use a comprehensive approach that takes into account multiple sources of information. In particular, evaluators must consider factors such as family history, history of speech and language difficulties, behavioral observations, and data from standardized tests of achievement and RtI programs. 

The assessment of cognitive processes determines the underlying cognitive and linguistic strengths and weaknesses; consideration of these factors influences the selection of both accommodations and interventions. Comprehensive evaluations provide information about individual learner differences that can then lead to the selection of targeted, individualized interventions (Decker et al. 2013). Tests are just tools; however, they help us understand development and behavior and determine what is needed to help an individual succeed in a school or work environment. As Meyer et al. (2001) explained, “Tests do not think for themselves, nor do they directly communicate with patients. Like a stethoscope, a blood pressure gauge, or an MRI scan, a psychological test is a dumb tool, and the worth of the tool cannot be separated from the sophistication of the clinician who draws inferences from it and then communicates with patients and professionals” (p. 153). Several decades ago, in a class lecture, Dr. Samuel Kirk was discussing test use and the importance of skilled observation and interpretation of behavioral data. To make his point, he used the following example: Imagine that you gave children “the board test.” This test would involve handing a child a hammer and 12 nails to put into a board. After watching many children do this task, a skilled evaluator would likely have insights into the children’s abilities to pay attention, their levels of impulsivity, and certain personality traits. 

Clearly, schools have an obligation to help any child who struggles to learn to read. Poor readers, regardless of their intellectual abilities or the reasons that contribute to their poor reading, benefit from evidence-based interventions (Siegel and Hurford 2019). Poor reading, however, is not synonymous with having dyslexia. Therefore, the overarching question addressed in this article remains: are tests of cognitive abilities useful in an evaluation for reading difficulties? If the intent is to only identify poor reading, the best answer is “no.” If, however, the intent is to explore the possibility that an individual has dyslexia, the best answer is “yes.”

Schneider and Kaufman (2017) observed that “… cognitive assessment helps professionals to understand the cause of the academic difficulty and that knowing the cause is helpful in selecting the correct course of action for treatment” (p. 13) and that the abilities measured by cognitive tests “… are too integral to academic difficulties for them to be, in the final accounting of the matter, irrelevant for helping individuals” (p. 18). Over eight decades ago, Stanger and Donohue (1937) explained, “If these tests will give us a basis from which we can start to understand a child’s difficulties, they will have justified the time spent on them. Anything which helps educators or parents to understand any phase of development or lack of development is of immeasurable value” (p. 189). For the assessment of dyslexia, when combined with other sources of data, tests of cognitive ability do just that. 

## Notes

1 The authors have argued that the PSW approach is unfeasible because it cannot be properly conducted without the purchase and use of at least nine high-quality instruments, including two measures for each factor of interest (Williams and Miciak 2018). This is not the approach taken when criticizing the efficacy of the model. Furthermore, rarely in the field would an evaluator use nine or more different tests. In a survey of 468 school psychologists, Shanock et al. (2022) found that 60% used between one and three instruments, whereas 8% reported using between seven and nine measures.

2 The selection of this subtest as the only measure of “working memory” in a study designed to evaluate the ability of PSW algorithms to predict differential treatment responses among learners with reading difficulties of various etiologies is indeed surprising, particularly as a coauthor noted that students with dyslexia tend to receive high scores. The subtest is derived from a relatively novel instrument that does not appear to be available for purchase and whose technical manual does not contain any statistical tables that can be used for profile analysis (Dehn 2011).

3 None of which constituted a complete battery or even a complete cluster; none of which involved the evaluation of verbal working memory; and none of which involved the evaluation of mathematics skills, rendering it difficult to establish diagnostic specificity.
